# Burden of respiratory syncytial virus disease in infants and the potential value of maternal immunization in Greece

**DOI:** 10.3389/fpubh.2025.1611483

**Published:** 2025-07-16

**Authors:** George Gourzoulidis, Argyro Solakidi, Eleftherios Markatis, Marios Detsis, Tania Siahanidou, Gabriel Dimitriou, Antonia Charitou, Charalampos Tzanetakos, Diana Mendes, Myrto Barmpouni

**Affiliations:** ^1^Health Through Evidence, Athens, Greece; ^2^Pfizer Hellas, Athens, Greece; ^3^First Department of Pediatrics, School of Medicine, National and Kapodistrian University of Athens, Athens, Greece; ^4^University General Hospital of Patras, University of Patras, Patras, Greece; ^5^Neonatal Department and Neonatal Intensive Care Unit, Rea Maternity Hospital, Athens, Greece; ^6^Pfizer Ltd, Tadworth, United Kingdom

**Keywords:** respiratory syncytial virus, maternal vaccination, Greece, infants, preventive strategy

## Abstract

**Objective:**

The objective was to assess the burden of respiratory syncytial virus (RSV) and evaluate the cost-effectiveness of maternal vaccination using the bivalent RSV prefusion F-protein (RSVpreF) vaccine to prevent RSV infections among Greek infants.

**Methods:**

A Markov model was adapted from the perspective of a public payer to simulate the health and economic outcomes of RSV from birth to 1 year of age. Key inputs for the model, including vaccine efficacy, utility values, epidemiological data, and direct medical costs [prices in euros (€), 2024], were obtained from official sources. Model main outcomes were medically attended RSV cases, RSV-related deaths, quality-adjusted life-years (QALY) gained, direct medical costs and incremental cost-effectiveness ratios (ICER).

**Results:**

The model analysis estimated that the annual number of RSV medically attended cases would be 21,935, with 22% requiring hospitalization, 32% managed in the emergency department (ED), and the remaining cases treated in outpatient settings. Furthermore, 11 RSV-related deaths were estimated. These cases represent a significant economic burden, with direct medical costs of ~€26 million. With a year-round maternal RSVpreF vaccination coverage of 19.5%, over 1,200 RSV medically-attended cases could be prevented annually. Vaccination benefits translated to 31 additional QALYs compared with no vaccination. Thus, the model analysis indicated that RSVpreF vaccination is a cost-effective strategy, resulting in an ICER of €8,280 per QALY gained compared to no vaccination.

**Conclusion:**

Administering maternal RSVpreF vaccination year-round can provide protection to infants against RSV from birth. From a payer perspective, maternal RSVpreF vaccination has been evaluated as a cost-effective alternative compared to no intervention, underscoring its value as a preventive strategy against RSV in Greece.

## Introduction

Respiratory syncytial virus (RSV) is a common pathogen that causes recurrent infections throughout a person's lifetime, with infants and chronically ill older adults most at risk of developing severe disease (1). RSV usually causes upper respiratory tract infections, but can also progress to acute lower respiratory tract infections (ALRTIs) (1). RSV is transmitted to infants primarily from their parents and other children, with the majority of cases acquired in a community setting (2–4).

RSV is the leading cause of viral bronchiolitis and pneumonia in children under 5 years of age, with infants under 6 months being at the highest risk for severe disease, including bronchiolitis and pneumonia, where respiratory distress is a key feature (5, 6). Globally, in 2019, RSV accounted for 33 million ALRTIs in children under 5 years, with 6.6 million cases occurring in infants under 6 months, equating to an incidence rate of 96.3 per 1,000 infants. The burden is particularly high in low- and middle-income countries (7). Furthermore, 45,700 ALRTI-related deaths in children under 6 months were attributed to RSV in 2019, with infants under 6 months accounting for over 50% of in-hospital RSV-related deaths among children under 5 years (7). Mortality and case fatality rates are highest in the first 3 months of life, making this a critical period of vulnerability (7–9). RSV causes more respiratory-related deaths than influenza and has a higher pneumonia-related case fatality ratio in children under 5 years (7, 10–12).

RSV leads to substantial clinical burden due to the high incidence of hospitalization, particularly in younger infants (7, 13, 14). Approximately 40% of the 3.6 million global RSV-associated hospitalizations are among children < 6 months, with infants in their first 3 months of life being most vulnerable and accounting for over 60% of hospitalizations in the first 6 months (7). Though prematurity and other chronic diseases increase risk of RSV-associated hospitalization, ~80% of hospitalizations are in healthy, term infants, emphasizing the need to protect all infants from RSV (15, 16). The burden of RSV is not limited to hospitalization and includes outpatient services as well as the management of recurrent respiratory infections; ~20% of infants hospitalized for RSV require readmission to hospital or Intensive Care Unit (ICU) with infants < 6 months accounting for up to 50% of all readmissions (17–19).

Moreover, RSV is associated with considerable direct healthcare use and costs, which has been estimated at ~€4.8 billion globally per year in children < 5 years of age (20). Direct health costs for RSV-associated ALRTI are particularly high in those aged < 6 months or with severe disease (18, 21). Hospitalization is the primary driver of RSV healthcare costs, with the cost of inpatient care influenced by disease severity and patient age, with the greatest burden incurred in those < 6 months of age (9, 18, 20–22). Caring for a child with RSV-associated ALRTI impacts a caregiver's ability to work to a greater extent than other respiratory infections (23).

The recently licensed bivalent RSV prefusion F protein vaccine (RSVpreF) shows promise in addressing an unmet medical need (24). This potential stems from its demonstrated efficacy, safety, and ability to provide protection against RSV in infants through the transfer of maternal antibodies generated in response to the vaccine, as demonstrated in a pivotal Phase III placebo-controlled clinical trial, the “MATernal Immunization Study for Safety and Efficacy” (MATISSE) (25).

Decision makers need to understand the potential health and economic outcomes of a maternal RSVpreF vaccination strategy to maximize the effective use of health care resources. Hence, the objective of this study was to evaluate the health and economic burden of RSV disease and the cost-effectiveness of maternal vaccination with RSVpreF for the prevention of RSV among infants in Greece.

## Materials and methods

### Target population

**Figure 1:**
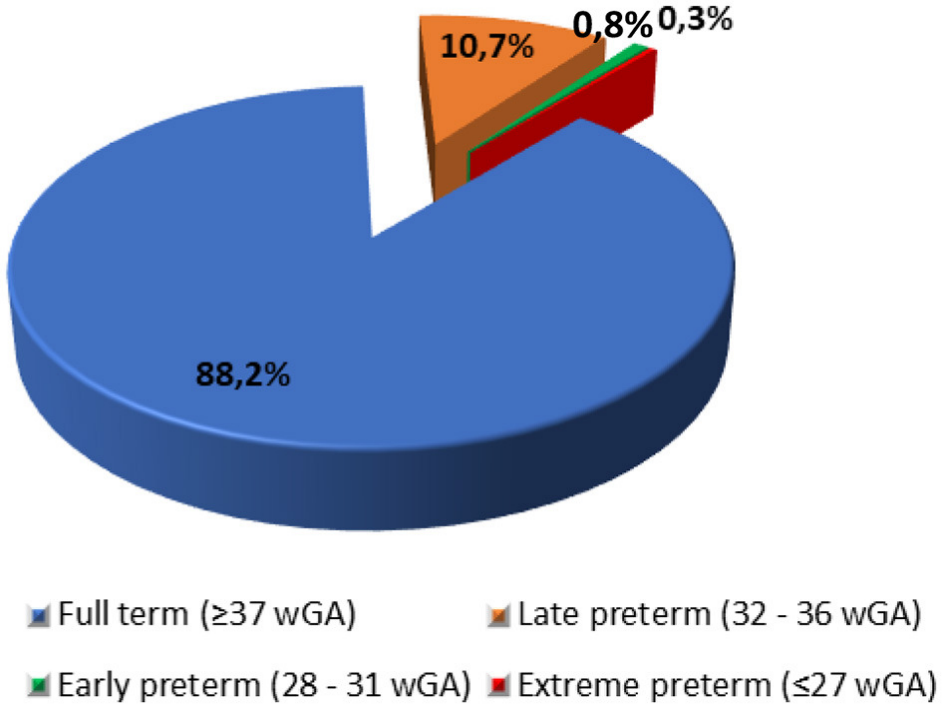
Distribution of births by term status. wGA, Weeks of gestational age. Source: Hellenic Statistical Authority (26).

The model population included liveborn infants (*n* = 76,541) born to 76,500 women during a 1-year period. The liveborn infants were characterized by gestational age in weeks (wGA) at birth, defined as: full term (≥37 wGA), late preterm (32–36 wGA), early preterm (28–31 wGA), and extreme preterm (≤ 27 wGA). Estimates of born infants (live and stillbirths), number of women giving birth and the distribution of births by term status (Figure 1) in a single year (2023) was provided by the Hellenic Statistical Authority (26).

### Model overview

The published model (27) utilizes a cohort-based framework and a Markov process to simulate health outcomes and economic costs associated with RSV infections in newborn infants from birth to 1 year of age. The model population was initially stratified based on weeks of gestational age (wGA) at birth. Infants were assumed to either receive protection against RSV through maternal vaccination or remain unvaccinated, representing the no-intervention scenario.

Expected clinical outcomes for infants were projected on a monthly basis (with a model cycle length of 1 month) using factors such as age, gestational age at birth (wGA), disease and fatality rates (which vary by age, wGA, and calendar month), and the mother's vaccination status, through the 1-year modeling horizon. Clinical outcomes included medically attended RSV cases, categorized by care setting [hospitalization, emergency department (ED), or outpatient visit (OV)], as well as RSV-related deaths requiring hospitalization. Infants whose mothers received RSVpreF were assumed to have a reduced risk of developing RSV, with the extent of risk reduction (including initial effectiveness and waning) influenced by the clinical setting (hospital vs. ED/OV), timing of maternal vaccination relative to birth, and the infant's wGA at birth. The risk of death from RSV and other causes (non-RSV) was modeled as age- and wGA-dependent. Moreover, the model incorporates a lifetime horizon to account for the long-term impact of premature RSV-related mortality on life expectancy, allowing for the estimation of total life-years (LYs) and quality-adjusted life years (QALYs) for the modeled cohort. The expected costs of medical treatment for RSV-LRTI were calculated based on unit costs associated with hospital, ED and OV, while vaccination costs, including vaccine unit cost and administration.

The base-case analysis was conducted from the perspective of the Greek public payer and incorporated only direct medical costs. A cost-effectiveness threshold of €44,000 per outcome gained or avoided was applied in the analysis [two times the Greek Gross Domestic Product (GDP) per capita]. A widely accepted assumption, drawn from various published studies, suggests that a health intervention can be deemed cost-effective if its incremental cost-effectiveness ratio (ICER) falls within the range of one to three times the country's GDP per capita (28–30). Additionally, a 3.5% annual discount rate was applied to both costs and outcomes in the analysis (31, 32).

### Model inputs and parameters

#### Disease incidence and mortality

In the absence of comprehensive national surveillance data on RSV burden in Greece, the annual incidence rates of RSV by month of age and by care setting (i.e., hospital, ED, and OV) were derived from a multidimensional real-world evidence study (33) (Table 1). This study was selected for its alignment with hospitalization patterns observed in Greece and was further validated by local clinical experts (neonatal pediatricians) from three major pediatric hospitals in Greece. These local clinical experts confirmed that RSV hospitalization rates extracted from the real-world evidence study (33) better reflect their real-world clinical experience than aggregated Europe estimates (7). A key consideration in our data selection was the variation in hospitalization practices across Europe. In many central European countries, hospital admissions for RSV require prior approval from general practitioners, whereas in Greece, infants are more readily admitted due to the absence of such a requirement and a lower threshold for hospitalization. A similar pattern has been observed in influenza-related hospitalizations, where Greek infants are hospitalized at rates above the Europe average (34, 35). Given these contextual factors, we determined that RSV hospitalization rates obtained from the real-world evidence study (33) serve as a reasonable proxy for Greece, as hospitalization rates in Greece are expected to be higher than the EU mean (14). To further validate our assumptions, we used the EU mean and explored the impact of alternative incidence estimates in a scenario analysis using RSV hospitalization rates from Del Riccio et al. (14).

**Table 1:** Incidence rates of respiratory syncytial virus by care setting and age, along with associated direct medical costs.

**Incidence rates for RSV (per 1,000)**				
**Age**	**Hospitalization**	**Emergency department**	**Outpatient visit**	**Source**
< 1 month	137.9	124.1	124.1	
1– < 2 months	164.3	166.3	168.3	(33)
2– < 3 months	94.3	101.4	102.9	
3– < 6 months	56.9	76.6	98.4	
6– < 12 months	34.9	85.5	146.0	
**Cost of RSV requiring hospitalization by age and term status** **(****44****)**				
Terms status	Age			
	< 1 month	1– < 2 months	2– < 6 months	6– < 12 months
Full term (≥37 wGA)	11,273 €	4,149 €	2,595 €	1,907 €
Late preterm (32–36 wGA)	13,441 €	11,273 €	4,149 €	2,595 €
Early preterm (28–31 wGA)	18,265 €	15,768 €	8,984 €	6,104 €
Extreme preterm (≤ 27 wGA)	31,460 €	23,575 €	15,768 €	11,864 €
**Cost of RSV treated in Emergency department by age and term status** ^*^				
All infants	203 €	203 €	203 €	203 €
**Cost of RSV treated in outpatient by age and term status** ^*^				
All infants	123 €	123 €	123 €	123 €

RSV, respiratory syncytial virus; wGA, weeks of gestational age.

^*^Source Greek Ministry of Health (43), official website of EOPPY (45) and local experts.

Age-specific relative rates of RSV based on term status (late, early, and extreme preterm compared to full-term infants) were calculated using data from the study by Rha et al. (36) and were assumed to be applicable across all care settings (Table 1). Due to the limited number of early and extreme preterm infants included in Rha et al.'s study, a combined relative rate of RSV was determined for these groups. The distribution of RSV cases across calendar months was determined using insights provided by local experts (Supplementary Figure S1).

RSV-associated mortality was based on hospitalization case-fatality rate (CFR), ~0.20–0.25 per 100 cases as reported in a published study (37) and estimates from local clinical experts. Furthermore, due to limited research on CFR related to RSV, the CFR for preterm infants was derived by applying a relative risk of death for preterm infants, as informed by a published study (11) and local clinical experts' estimation. At this point, it should be mentioned that it was assumed infants with RSV who were treated in the outpatient setting were not considered to be at risk of disease-related death. Moreover, the general infant mortality rate (per 1,000 live births, death due to non-RSV causes) was obtained from the Hellenic Statistical Authority (26).

#### Maternal vaccination effectiveness and coverage

**Figure 2:**
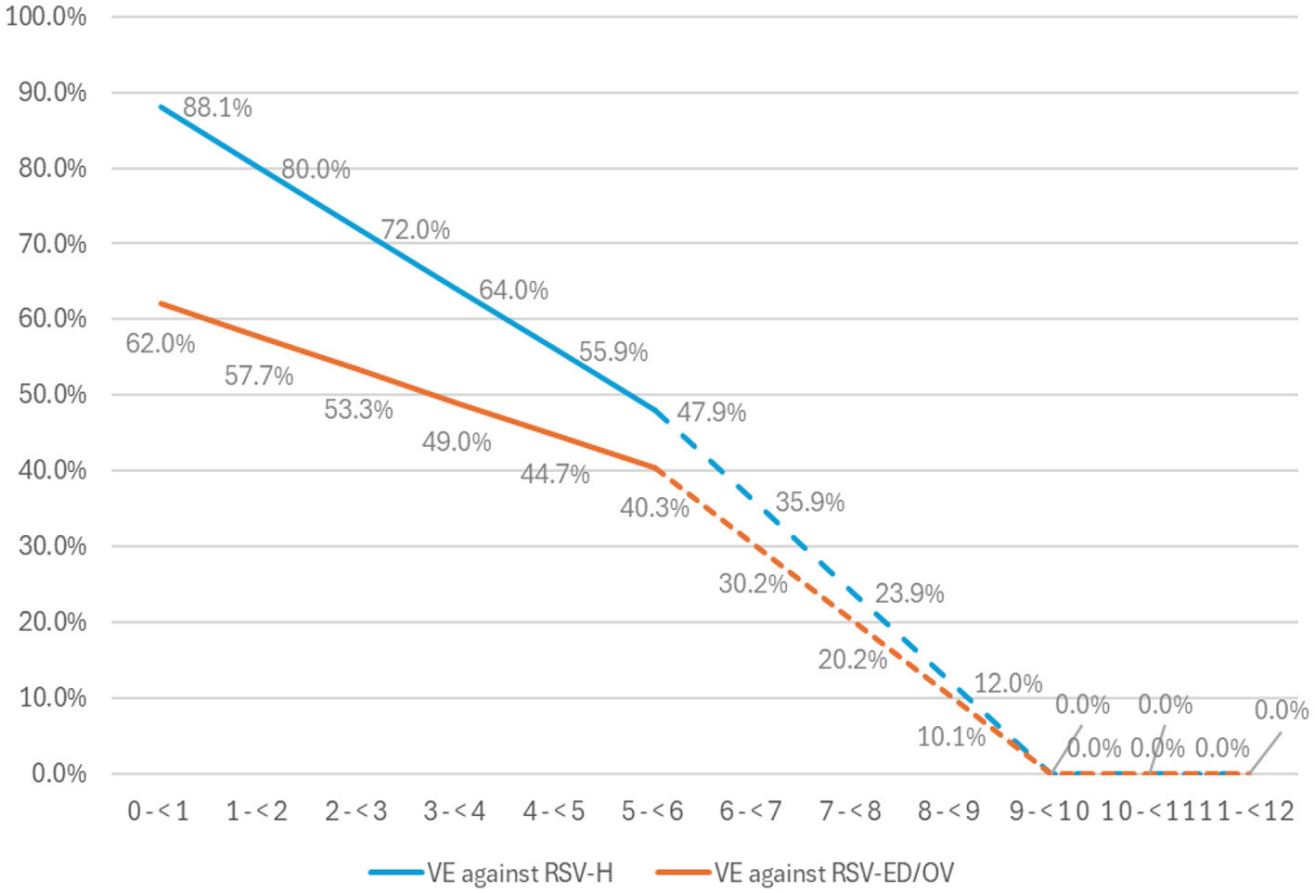
The effectiveness of the vaccine (VE) against RSV-LRTI requiring hospitalization (RSV-H) and RSV-LRTI treated in the emergency department or outpatient visit for full term and late preterm infants born at least 2 weeks after maternal vaccination. ED, emergency department; OV, outpatient visit; RSV, for respiratory syncytial virus. Observed RCT VE data in solid lines and extrapolated data in dashed lines.

Setting-specific vaccine effectiveness (VE) estimates were derived from the cumulative efficacy data for the primary endpoints of the MATISSE trial (25). The efficacy of the MATISSE study against severe RSV-positive medically attended lower respiratory tract illness has been comprehensively presented in previous related published studies (27, 38, 39). At this point, it is important to emphasize that vaccine efficacy (VE) was assumed to decline linearly from 6 months post-vaccination to 0% by 9 months, as illustrated in Figure 2.

Moreover, the coverage of maternal vaccination (RSVpreF) was assumed to be 19.5% based on values for influenza vaccination of pregnant women as reported in a recent Greek study (40) and was assumed to be constant across calendar months. The vaccine administration window was assumed to be between 24 and 36 weeks consistent with EMA's regulatory approval, and the distribution of RSVpreF administration by fetal wGA was informed by local experts.

#### Utilities data

A utility value of 1 was assumed for infants without RSV-LRTI. For those with RSV-LRTI, utility values during a 14-day illness period were set at 0.59 for RSV-related hospitalization and 0.84 for RSV cases treated in ED or OV settings (41). The corresponding QALY losses were estimated to be 0.0157 for infants experiencing RSV-related hospitalization and 0.0061 for those treated in ED/OV settings (41). These QALY losses were assumed to remain consistent across all term statuses (41). For individuals aged ≥1 year, utility values were derived from reference population norms in a published study (42) or children aged 1–17 years, utility values were estimated through linear interpolation between values for 1-year-olds and adults aged ≥18 years.

#### Costs inputs

Cost inputs included vaccination expenses and direct medical costs attributable to RSV for episodes of RSV-related hospitalization (RSV-Hospital), RSV-ED, and RSV-OV, categorized by age and term status.

The bivalent RSVpreF unit cost per dose of €205.98 was extracted from the Greek Ministry of Health (43). Following local clinical practice, it was assumed that all pregnant women would need a healthcare professional for the administration of vaccine, hence, the cost of a physician visit was charged (€10 per visit).

The age- and term-specific direct medical costs of RSV hospitalizations were derived from DRG tariffs set by the Greek Ministry of Health (44). Costs for non-hospitalized RSV cases, including ED and OV, were estimated based on resource use reported by local experts and unit costs from the National Organization For Health Care Services (EOPYY) official website (45), and Greek Ministry of Health (43). All unit costs correspond to the year of the analysis (2024, €; Table 1).

## Model sensitivity and scenario analyses

To address statistical uncertainties in several key parameters, both deterministic sensitivity analyses (DSA) and probabilistic sensitivity analyses (PSA) were conducted. A one-way DSA was performed to identify the key drivers of the model and assess areas of uncertainty. For variables where sensitivity parameters were unavailable, upper and lower bounds were tested using a ±25% variation from the mean value. DSA were performed for the following variables: vaccination effectiveness, disutilities, RSV hospitalization rate, and cost input. The impact of joint parameter uncertainty was explored by PSA. Typical probability distributions were used in the analyses (46). A total of 1,000 simulations were conducted to generate a distribution of incremental results, providing an estimate of the overall parametric uncertainty surrounding the cost-effectiveness findings.

In order to test key uncertainty not addressed in either the DSA or PSA, several specific scenario analyses were conducted. More specifically, (1) vaccination administration window between 32 and 36 weeks of gestation, (2) seasonal administration of vaccination (September to January), (3) vaccination between 32 and 36 weeks of gestation and seasonal administration (September to January), (4) vaccination of all pregnant women (vaccine coverage of 100%), and (5) Incidence rates of RSV hospitalization from Del Riccio et al. (14) were applied to assess any impact on the overall results.

## Results

### Base case results

The model analysis estimated that the annual number of RSV medically attended cases in Greece would be 21,935, with 22% requiring hospitalization, 32% managed in the ED, and the remaining cases treated in OV. Furthermore, 11 RSV-related deaths were estimated. These RSV-cases represent a significant economic burden, with direct medical costs of ~€26 million (Table 2).

**Table 2:** Base case model results.

**Parameters**	**Maternal vaccination**	**No vaccination**	**Incremental**
**Health outcomes**			
**No. of RSV medically-attended cases**			
Hospital	4,401	4,884	−484
Emergency department	6,820	7,155	−335
Outpatient visit	9,507	9,896	−389
Total	20,727	21,935	−1,208
No. of RSV-related deaths	10	11	−1
Total quality-adjusted life years	1,863,469	1,853,558	31
Total life years	2,064,605	2,053,508	21
**Economic outcomes (in millions)**			
Direct cost of vaccination (€)	3.15	–	3.15
Direct RSV medical care cost (€)	22.98	25.87	−2.89
Total cost (€)	26.13	25.87	0.26
**Cost-effectiveness analysis**			
Incremental cost-effectiveness ratio per quality-adjusted life year gained (€)			8,280
Incremental cost-effectiveness ratio per life year gained (€)			12,082
Incremental cost-effectiveness ratio per RSV hospitalized case avoided (€)			528

RSV, respiratory syncytial virus.

Year-round RSVpreF vaccination with 19.5% coverage was projected to reduce hospitalizations by 484 cases, ED encounters by 335 cases, OV by 389 cases, and RSV-related deaths by 1 over a 1-year period. The effectiveness benefits associated with RSVpreF vaccination translate into this strategy accruing 31 QALYs compared to no vaccination (Table 2). The model analysis demonstrated that RSVpreF vaccination is a cost-effective strategy. It estimated ICERs of €12,082 per LY gained, €8,280 per QALY gained, and €528 per RSV-related hospitalization avoided, compared to no vaccination (Table 2).

### Sensitivity and scenario analyses results

The DSA results demonstrated that the base case model outcomes were robust to variations in clinically reasonable parameter inputs. The model was most sensitive to changes in the efficacy of the RSVpreF vaccine, the hospitalization rate associated with RSV, and the cost of the RSVpreF vaccine (Table 3).

**Table 3:** Deterministic sensitivity analyses results.

**Parameter**	**Lower bound −25%**	**Upper bound +25%**
Disease incidence	ICER/QALY (€)	ICER /QALY (€)
RSV hospitalization	39,197	Dominant
RSV ED	8,964	7,618
RSV outpatients visit	8,823	7,757
**Mortality**		
General infant mortality	8,231	8,329
Case-fatality due to RSV hospitalization	9,790	7,174
**Effectiveness**		
Maternal vaccine	42,253	Dominant
**Cost of vaccination**		
Maternal vaccine	Dominant	32,579
**Direct cost of disease**		
RSV hospitalization	30,773	Dominant
RSV ED	8,818	7,742
RSV outpatients visit	8,657	7,902
**Utilities data**		
Healthy infant utility	8,321	8,239
Disutility due to RSV hospitalization	8,813	7,808
Disutility due to RSV ED	8,416	8,148
Disutility due to RSV outpatients visit	8,439	8,127

ICER, incremental cost-effectiveness ratio; QALY, quality-adjusted life year; RSV, respiratory syncytial virus; ED, emergency department; Dominant, improved outcomes in terms of QALY gain with reduced total costs, i.e., more effective and less costly than no vaccination.

**Figure 3:**
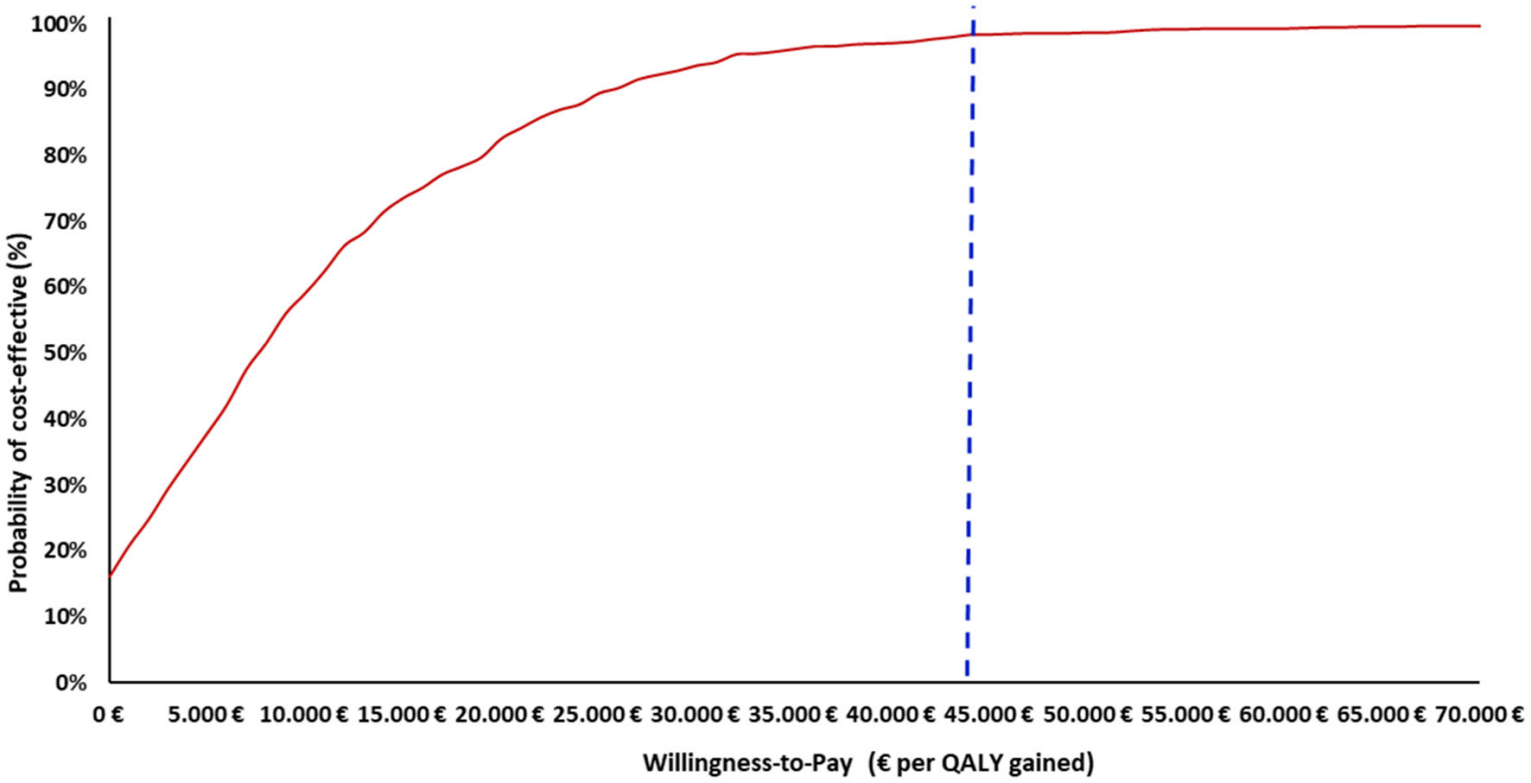
Cost–effectiveness acceptability curve of maternal vaccination strategy vs. no vaccination strategy. QALY, Quality-adjusted life year. The *x*-axis indicates the WTP thresholds (€), while the y-axis represents the probability that the vaccination strategy is cost-effective at each threshold level. The vertical dashed line marks the cost-effectiveness threshold of €44,000 per QALY gained.

Furthermore, the PSA results indicated that, at an assumed cost-effectiveness threshold of €44,000 per QALY gained, the maternal vaccination had a 98% probability of being a cost-effective option compared to no vaccination (Figure 3). Additionally, across all scenario analyses, the maternal vaccination consistently remained a cost-effective strategy compared to no vaccination staying below the cost-effectiveness threshold, defined as one (€22,000) to three (€66,000) times the GDP per capita of Greece (Table 4).

**Table 4:** Scenario analyses results.

**Description**	**Maternal vaccination vs. No vaccination**
**1) Vaccination administration window between 32 and**	
**36 weeks of gestation**	
Difference in costs (in millions, EUR)	€0.45
Difference in QALYs	26
Incremental cost-effectiveness ratio per QALY gained	€17,527
**2) Seasonal administration of vaccination**	
Difference in costs (in millions, EUR)	–€0.39
Difference in QALYs	19
Incremental cost-effectiveness ratio per QALY gained	Dominant
**3) Vaccination between 32 and 36 weeks of gestation and**	
**seasonal administration**	
Difference in costs (in millions, EUR)	–€0.32
Difference in QALYs	21
Incremental cost-effectiveness ratio per QALY gained	Dominant
**4) Vaccination of all pregnant women (100% coverage)**	
Difference in costs (in millions, EUR)	€1.31
Difference in QALYs	159
Incremental cost-effectiveness ratio per QALY gained	€8,228
**5) Incidence rates of RSV hospitalization from**	
**Del Riccio et al**. **(****14****)**	
Difference in costs (in millions, EUR)	€1.32
Difference in QALYs	22
Incremental cost-effectiveness ratio per QALY gained	€60,906

QALY, quality-adjusted life year; Dominant, improved outcomes in terms of QALY gain with reduced total costs, i.e., more effective and less costly than no vaccination.

## Discussion

This study suggests that maternal vaccination with RSVpreF would be a cost-effective intervention to prevent RSV infections among infants, reduce hospitalizations and alleviate strain on medical resources. Assuming a year-round RSVpreF maternal vaccination coverage of 19.5%, >1,200 RSV medically-attended cases could be prevented annually with 31 additional QALYs gained and ICERs of €12,082 per LY gained, €8,280 per QALY gained and €528 per RSV hospitalized case avoided compared to no vaccination. Sensitivity analysis revealed robustness of the base-case findings to changes in input parameters and assumptions with maternal RSVpreF vaccination being cost-effective (and even cost saving) vs. no vaccination in all sensitivity and scenario analyses.

Our findings are consistent with those presented in the previously conducted studies of maternal vaccination with RSVpreF. More specifically, a cost-effectiveness study conducted in Spain (27) from a payer perspective showed that maternal immunization with RSVpreF vaccine was a dominant alternative (more effective and less costly) compared with a no vaccination strategy. Similar findings were reported for Canada (47), which showed that from a societal and payer perspective the maternal vaccination with RSVpreF was cost-effective compared to no vaccination at the cost-effectiveness threshold of $50,000 per QALY gained. Moreover, a multi-country study that was conducted in Europe (48) showed that from the payer perspective, maternal vaccination with RSVpreF compared to no vaccination strategy was cost-saving in Scotland, and cost-effective at threshold of €20,000–30,000 per QALY gained in England, Finland and Denmark.

It should be emphasized that the value of maternal vaccination to protect infants from RSV suggests multifaceted benefits, from direct health protection to the mother and infant to long-term societal impacts (49, 50). The RSV vaccine may offer protection to infants during their most vulnerable developmental stages and has the potential to reduce RSV-related long-term consequences (50). Additionally, it could contribute to herd immunity, potentially offering some level of protection to those who cannot be vaccinated (51, 52). Apart from the humanistic and clinical benefit, vaccination may reduce healthcare costs by preventing disease outbreaks and reduce the need for medical treatments and hospitalizations (51–53). Furthermore, protection against RSV in infancy ensures healthier childhood development, reduces long-term health complications, and promotes a better quality of life (51, 53).

Overall, vaccination is not only a successful health intervention, but also an effective investment in healthcare system. Recent studies showed that for every €1 invested in routine childhood immunization resulted €3 in cost savings from a societal perspective for national pediatric immunization programs (54, 55). Hence, it becomes clear that vaccination intervention such as RSV immunization is a sound decision, promoting public health by reducing the overall incidence of the virus and alleviating the burden on healthcare systems (56). Thus, RSV maternal vaccination to protect infants is a vital strategy for improving health outcomes and optimizing healthcare delivery in Greece.

Even though an established methodology was used in this study, some potential limitations should be acknowledged. First, in the absence of local RSV-specific incidence data for Greece, incidence rates were derived from a published study and validated by local clinical experts with extensive experience in managing respiratory infections. While we selected published study real-world data as the primary source due to its alignment with hospitalization patterns observed in Greek clinical settings, we recognize that using data from another country introduces potential generalizability concerns. This selection was validated by local clinical experts (neonatal pediatricians), who confirmed that Spanish hospitalization rates closely mirror their clinical experience. Additionally, differences in hospitalization criteria between Greece and central Europe—where Greek infants are admitted more readily—support the rationale that Spanish hospitalization rates may better approximate the burden in Greece compared to aggregated EU estimates. To address concerns about potential overestimation, we conducted a sensitivity analysis using hospitalization estimates from Del Riccio et al. (14), a systematic review providing RSV hospitalization rates for multiple EU countries, including Greece. This additional analysis allowed us to test more conservative assumptions and assess the robustness of our cost-effectiveness findings under different hospitalization scenarios. Moreover, to account for potential variability, a sensitivity analysis was conducted, exploring variations of ±25% in disease base case incidence rates. The results demonstrated that maternal vaccination remained a cost-effective option, underscoring the robustness of the model outputs. The primary findings were consistent across a wide range of parameter values. However, our findings should be interpreted with caution, and we emphasize the urgent need for high-quality, Greece-specific RSV surveillance data to strengthen future evaluations. Second, the current model does not capture the potential direct effects of vaccination against RSV among vaccinated pregnant individuals either before or after giving birth since maternal vaccination efficacy among pregnant individuals was not an outcome in the MATISSE phase 3 trial. Future research should explore this dimension, as it may further enhance the overall value proposition of maternal RSV vaccination Third, in the absence of local data, RSV-associated mortality in our model was estimated based on published studies and input from local clinical experts. However, a series of sensitivity analyses demonstrated that the model outcomes are robust, as the main results remained consistent across a wide range of parameter values. Fourth, our analysis was conducted from the perspective of the public payer and, therefore did not incorporate indirect societal costs such as caregiver absenteeism, loss of productivity, or long-term respiratory sequelae associated with severe RSV infections (e.g., wheezing, asthma). While not modeled explicitly, the inclusion of these indirect costs in future studies may further strengthen the economic case for maternal RSV vaccination, given the well-documented burden of RSV on families and the healthcare system.

## Conclusions

The present study found that administering maternal RSVpreF vaccination year-round can provide protection to infants against RSV from birth. From a payer perspective, maternal RSVpreF vaccination has been evaluated as a cost-effective alternative compared to no intervention, underscoring its value as a preventive strategy against RSV in Greece.

## Data Availability

The original contributions presented in the study are included in the article/Supplementary material, further inquiries can be directed to the corresponding author.
