# Supplementary material for: Burden of respiratory syncytial virus disease in infants and the potential value of maternal immunization in Greece
Source: Front Public Health. 2025 Jul 16;13:1611483. doi: 10.3389/fpubh.2025.1611483 (PMC12307291; doi:10.3389/fpubh.2025.1611483)
Supplement: Supplementary file 1 [file Data_Sheet_1.pdf]

Supplementary material

Figure 1: Distribution of respiratory syncytial virus cases by calendar month

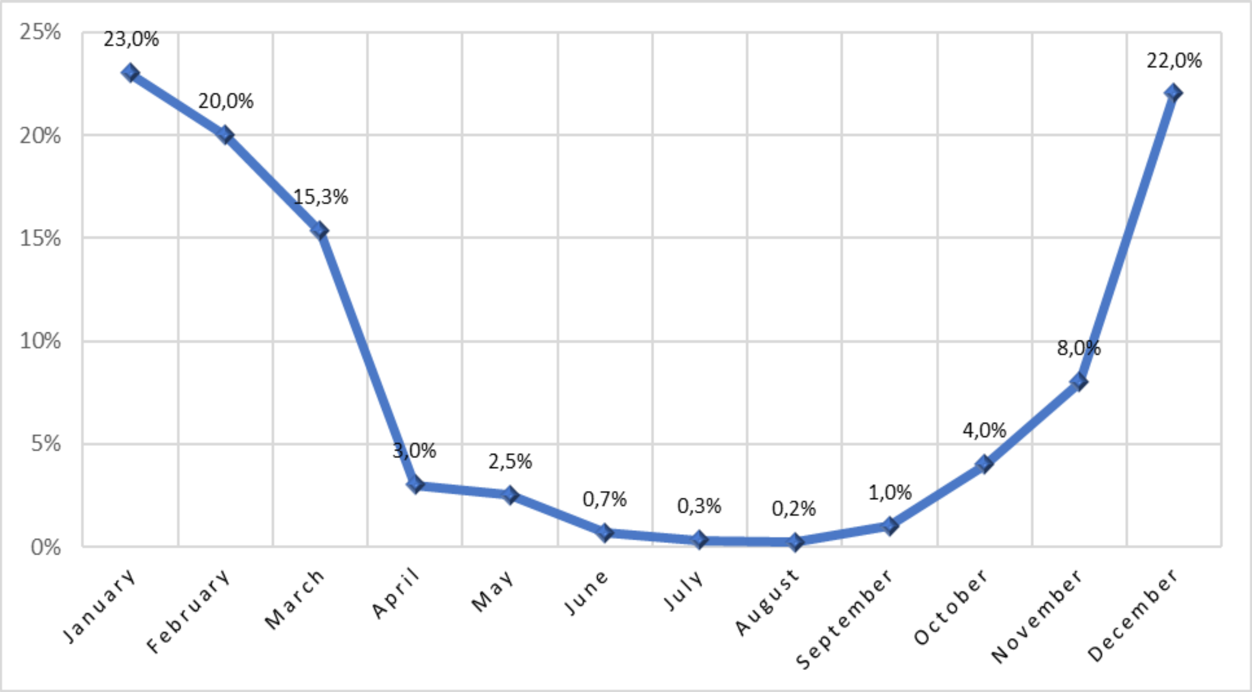

Source: Local clinical experts
